# Influence of active versus passive parental presence on the behavior of preschoolers with different intelligence levels in the dental operatory: a randomized controlled clinical trial

**DOI:** 10.1186/s12903-021-01781-z

**Published:** 2021-08-28

**Authors:** Thiyezen Abdullah AlDhelai, Amani Mohamed Khalil, Yasmine Elhamouly, Karin M. L. Dowidar

**Affiliations:** 1 Department of Orthodontics and Pediatric Dentistry, College of Dentistry, Qassim University, Buraydah, Saudi Arabia; 2 Department of Pediatric Dentistry and Dental Public Health, Faculty of Dentistry, Alexandria University, Alexandria, Egypt; 3 Pediatric and Community Dentistry Department, Faculty of Dentistry, Pharos University in Alexandria, Alexandria, Egypt

**Keywords:** Behavior modification, Intelligence quotient, Parental active/passive presence, Preschool children, Stanford Binet intelligence scale

## Abstract

**Background:**

Dental fear and anxiety still pose the most common factors proposed for the child’s negative behavior in the dental operatory. Intelligence has an impact on the children’s communication, feelings, and responsiveness to dental situations. The benefits of parental presence on reinforcing the child’s behavior during dental treatment are still debatable. This study aimed to assess the effect of parental active versus parental passive presence techniques on the overall behavior of preschool children with different intelligence levels.

**Methods:**

This randomized controlled trial was conducted from December 2017 to August 2019. It recruited 150 healthy children, 3–6-year-old, with no history of previous dental pain/treatment, and intelligence quotient level of 70– ≤ 110 stratified into 3 equal groups (high, average, low). In the first visit, each IQ group was randomly divided into test (PAP) and control (PPP) groups. In the second visit, dental fear was assessed before preventive intervention, the test groups were then managed using parental active presence technique, while the control groups were managed using parental passive presence technique. The overall behavior was assessed at the end of the visit. Data was analyzed using Chi-square test and logistic regression analysis.

**Results:**

The parental active presence technique had significant effect on children with high and low intelligence quotients. There were significantly higher odds of positive behavior in high than low intelligence quotient children, (OR 4.08, 95% CI 1.43, 11.67, P = 0.01). The parental active presence technique had significantly higher odds of positive behavior than the parental passive presence technique, (OR 4.08, 95% CI 1.71, 9.76, P = 0.002).

**Conclusions:**

The parental active presence technique had positively influenced the children’s overall behavior irrespective of their intelligence levels. This trial was retrospectively registered, trial identifier number: NCT04580316, 8/11/2020.

**Supplementary Information:**

The online version contains supplementary material available at 10.1186/s12903-021-01781-z.

## Background

Understanding children’s development and behavior are crucial issues in pediatric dentistry [1]. Physical, social, emotional, and cognitive/intellectual developments are integrated and influence one another [2]. Different predictors were demonstrated for children’s negative behavior in the dental setting. A strong correlation was proposed between dental fear and children’s behavior so that it could be used as a behavior indicator in the dental setting [3, 4]. Moreover, it was noted that the child’s age was significantly related to his behavior; in which more negative behavior should be expected from younger children. Hence, age would be considered an influential factor in the overall behavior of children during dental treatment. Furthermore, female children were proposed to show higher levels of dental fear and thus negative behavior than that of male children [4]. Additionally, previous unpleasant dental experiences have been shown to influence the display of children’s negative behavior in the dental clinic [1]. However, the exact reason why some children well behave in the dental operatory while others do not despite being under the same conditions is still not obvious. Therefore, it seems that another hidden factor, probably the cognitive factor, needs to be counted [5]. The Cognitive factor plays a major role in dental fear and anxiety (DFA) [6]. The correlation between children's intelligence and DFA, as well as their behavior in the dental clinic is a sensitive issue [7–11]. Children’s behavior represents a great challenge in dental practice, as safe and effective treatment requires the shaping of the child and his parents’ behaviors towards dental care. Hence, the term ‘behavior guidance’ has been developed. It entails that the dentist/dental team interact with the patient and the parents to allay DFA in order to establish a good rapport. This is needed for providing quality treatment and for encouraging a positive attitude towards oral health care [12].

Behavior management techniques must be integrated into an overall behavior guidance approach customized for each child. One of the proposed behavior management techniques is the parental presence/absence technique [12]. However, the benefits of this technique to the children during dental treatment are  still conflicting [13]. Some studies have demonstrated that parental presence/absence does not affect the child’s anxiety or cooperation during dental sessions [14, 15].

On the other hand, it was reported that some dentists prefer to have the parents outside the operating room, as their presence may complicate communication with the child, or they may exhibit anxiety themselves [12]. Nevertheless, parental presence may be implemented to improve the children’s behavior, especially for the young ones and those with limited cooperation, by helping them reduce their anxiety and cope with the new environment [16, 17].

Parental presence might be active or passive during dental treatment [18]. Involving the parent as a passive yet silent helper can provide a relaxing atmosphere without unnecessary interference [14]. However, if the parent is properly educated, he/she could actively act as a valuable adjunct in establishing rapport between the child and the dentist during treatment [18, 19].

Intelligence has a significant impact on children’s communication, feelings, and responsiveness to dental situations [20]. Limited studies have dealt with the association between children's intelligence and their overall behavior in the dental clinic. The influence of parental presence/absence on the children’s behavior and fear during dental treatment has been abundantly evaluated in the literature [21]. However, to our knowledge, no evidence of research has investigated the effect of parental active versus passive presence technique on children’s behavior in the dental setting. Therefore, this randomized controlled clinical trial was conducted to assess the effect of parental active and passive presence techniques (PAP/PPP) with tell-show-do (TSD) on the behavior of preschool children with different levels of intelligence. The null hypothesis was that there is no difference between the effect of PAP and PPP on the behavior of preschool children with different intelligence levels.

## Methods

### Study design and study setting

This parallel design randomized controlled clinical trial was set according to the CONSORT statement (Additional file 1) [22]. It was conducted in the outpatient clinic of the Pediatric Dentistry Department, Faculty of Dentistry, Alexandria University, Egypt, from December 2017 to August 2019. The PICO question was: In preschool children with different intelligence levels (P) how does parental active presence technique (I) compared to parental passive presence technique (C) affect the overall behavior (O) during preventive dental treatment?

### Sample size calculation

The sample size was based on assuming 5% alpha error, 20% beta error, allocation ratio between test and control groups of 1:1, and probability of positive behavior in the control group of low intelligence quotient (IQ) = 0.25 [23]. An estimation was made of the probability of positive behavior in the test group with low IQ to have positive behavior as healthy children = 0.87 [24]. So, to detect the difference between control and test groups, it was calculated that 9 children per group would be needed. [25] To ensure adequate power, we estimated a reduction in the difference between test and control groups regarding probabilities of positive behavior of 50% with the calculated required number = 24. This was increased to 25 children per group to account for non-completion. Thus, the total required number of children = number of IQ groups X number of intervention groups X number per intervention group = 3 X 2 X 25 = 150 children.

### Participants and ethics approval

Participants enrolled in this study were healthy children, 3–6 years old, requiring preventive treatment, with no history of previous dental treatment or dental pain, and with IQ levels in the range of 70– ≤ 110. Children were excluded if they have pain, multiple dental problems, and history of medical and/or psychological problems. The study protocol (Additional file 2) was approved by the Research Ethics Committee, Faculty of Dentistry, Alexandria University, Egypt, (IRB 00010556)-(IORG 0008839). It was retrospectively registered at (https://clinicaltrials.gov), trial identifier number: NCT04580316, (8/11/2020). Prior to commencement, the study purpose, risks, and benefits were explained to the parents and a signed informed consent was obtained from the parents of all participants involved in the study.

### Randomization and allocation concealment

Children were stratified based on their IQ level into 3 groups: high, average, and low IQ groups. In each group, children were randomly and equally allocated according to intervention into test (PAP) and control (PPP) groups. Randomization was performed by a trial independent person using a computer random number generator. Randomization sequence in blocks of 2 was created using random allocation software version 1.0.0 [26]. The allocated group was written on a piece of paper that  was folded and enclosed in a sealed envelope that carried the child’s name on its cover. At the time of intervention, an assistant opened the envelope, and identified the group to which the child was assigned.

### Intervention and study outcomes

Prior to the study, intra-examiner reliability was assessed to ten children not participating in the study for the application of the facial image scale (FIS) and Frankl behavior rating scale (FBRS). These children were re-evaluated after five days. The kappa values obtained were 0.71 and 0.73 respectively [27].

In the first visit, visual screening and comprehensive history taking were carried out to identify children who fulfilled the inclusion criteria. Children were stratified into 3 equal groups (50 children/group) by the same trained and certified operator according to their IQ level as follows: high IQ (HIQ) with scores above 110, average IQ (AIQ) with scores of 90–110, and low IQ (LIQ) with scores of 70–89. The IQ was measured using the Arabic version of the Stanford Binet intelligence scale, fourth edition (SB-IV) [28]. It is standardized for Arabic Egyptian norms and includes a wide verbal reasoning item that can lead to a valid strong verbal IQ [8]. The SB-IV is a flexible, easy-to-implement collection of tests presented in a form of photos, different colored cubes, cubic's blossom, beads, paper tests, and some guiding books. The test is grouped into four area scores: verbal reasoning, abstract/visual reasoning, quantitative reasoning, and short-term memory reasoning. Eight sub-tests (abbreviated test battery) were selected from the total 15 sub-tests (full test battery) of SB-IV scale according to the age group, and they were listed as follows: vocabulary (V); comprehension (Com); absurdities (Ab); pattern (P); copy (Cop); quantitative (Q); bead memory (BM); and memory for sentence (MS) [20, 29] The test was made by the researcher himself in a quiet closed room. Administration of the SB-IV scale typically takes between 30–90 min, and the parent of each child has attended the examination passively. Age-appropriate oral hygiene instructions were provided to the patient and his accompanying parent at the end of the visit.

In the second visit, dental fear (DF) was assessed before the intervention using FIS [30]. This scale consists of a row of five faces ranging from a very happy face to a very unhappy one. Children were asked to point at the face they mostly like at that moment. The face was scored by giving a value of (1) to the most positive effect face and (5) to the most negative face. Faces with values (1, 2) indicated low DF, value (3) indicated moderate DF, and faces with values (4, 5) indicated high DF [30].

In each IQ group, random allocation was performed so that 25 children were managed during intervention using PAP + TSD (test group), while the other 25 children (control group) were managed using PPP + TSD [31]. The PAP technique was implemented in this study in which the parents were allowed to stand close to their children, do handholding, eye contacting and help in explaining the dentist’s instructions [16]. Conversely, in the PPP technique, parents were instructed to sit silently in the dental clinic behind the patient with no eye contact, and without a spoken word only to reassure their children. Non-pain provoking preventive measures were implemented including dental prophylaxis (Alpha-Pro ® preventives Prophylaxis Past, Dental Technologies, Hamlin Avenue, Lincolnwood, Illinodis. USA) followed by fissure sealant (bioseal ® Pit and Fissure Sealant, Biodinamica, Madrid. Spain), and/or topical fluoride application (Sorbet ® Fluoride gel, Keystone Industries, Hollywood Avenue, Cherry Hill. USA).

### Outcome assessment

The intervention was video recorded, and a blinded examiner evaluated the children’s overall behavior in both groups using FBRS [17, 19] Rating 1 (– –) was given to the most negative child behavior and rating 4 (+ +) to the most positive child behavior. The FBRS of (3, 4) were re-coded to positive behavior, while scores (1, 2) were recoded to negative behavior.

### Statistical analysis

Descriptive statistics were calculated as frequencies and percentages. The comparison between test and control groups was done using Chi-squared test. Logistic regression models were used with adjustment for confounding factors (age, gender, and fear level) for better models' fit. The models assessed the effect of PAP and PPP and IQ levels on the outcome (behavior dichotomies into positive and negative behaviors). Wald Chi-square, their p values, estimates (OR), their 95% confidence intervals (CI), and model adjusted Negelkerke R^2^ values were calculated. Intention to treat analysis was applied. Statistical analysis was done using SPSS version 17.0 (SPSS Inc., Chicago, Ill., USA). The significance level was set at 5%.

## Results

**Fig. 1:**
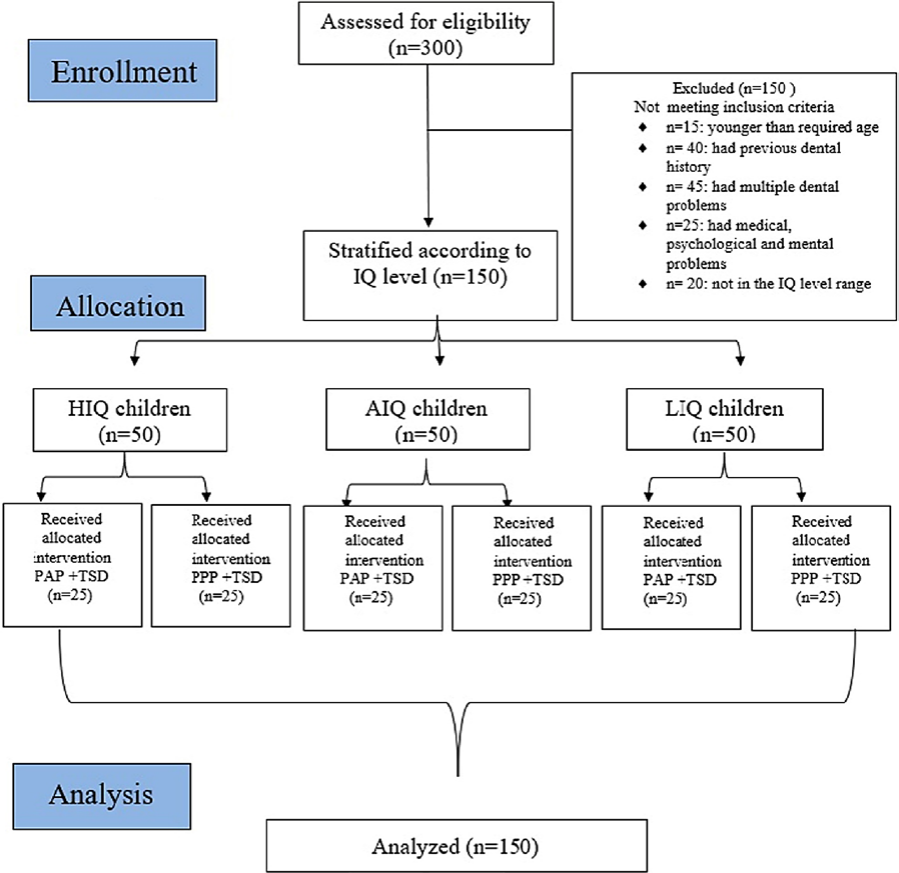
CONSORT flow diagram of the study participants

Out of 300 screened children, 150 were recruited (Fig. 1). The age range was divided into three groups. The 1st group ranged from (3- < 4) years, with 26.7% and 24% of the children in the PAP and PPP groups, respectively. The 2nd group ranged from (4- < 5) years, with 22.7% and 33.3% of the children in the PAP and PPP groups, respectively. The 3rd group ranged from (5–6) years, with 50.7% and 42.7% of the children in the PAP and PPP groups, respectively. At baseline, there was no statistically significant difference in the distribution of children in the three age groups, (P = 0.34) nor in the intelligence quotient levels, (P = 1.000) among the test and control groups. More male children (60%) were presented in the PAP group while more female children (42.7%) were presented in the PPP group with no statistically significant difference, (P = 0.74) between the study groups. More fearful children were presented in the PAP group (62.7%) compared to the PPP group (48.0%) with no significant difference in the FIS scores (P = 0.71) between the test and control groups (Table 1).

**Table 1:** Distribution of demographic variables, intelligence quotient, and fear levels among study groups before intervention

Factors	PAP n (%)	PPP n (%)	P value
	N = 150 children		
*Age*			
3–< 4 years	20 (26.7%)	18 (24%)	0.34
4–< 5 years	17 (22.7%)	25 (33.3%)	
5–6 years	38 (50.7%)	32 (42.7%)	
*Gender*			
Male	45 (60.0%)	43 (57.3%)	0.74
Female	30 (40.0%)	32 (42.7%)	
*Intelligence quotient*			
HIQ	25 (33.3%)	25 (33.3%)	1.000
AIQ	25 (33.3%)	25 (33.3%)	
LIQ	25 (33.3%)	25 (33.3%)	
*Fear*			
Unfearful	28 (37.3%)	39 (52.0%)	0.71
Fearful	47 (62.7%)	36 (48.0%)	

^*^ Statistically significant at P < 0.05

*HIQ* high intelligence quotient group, *AIQ* average intelligence quotient group, *LIQ* low intelligence quotient group, *PAP* parental active presence, *PPP* parental passive presence

**Fig. 2:**
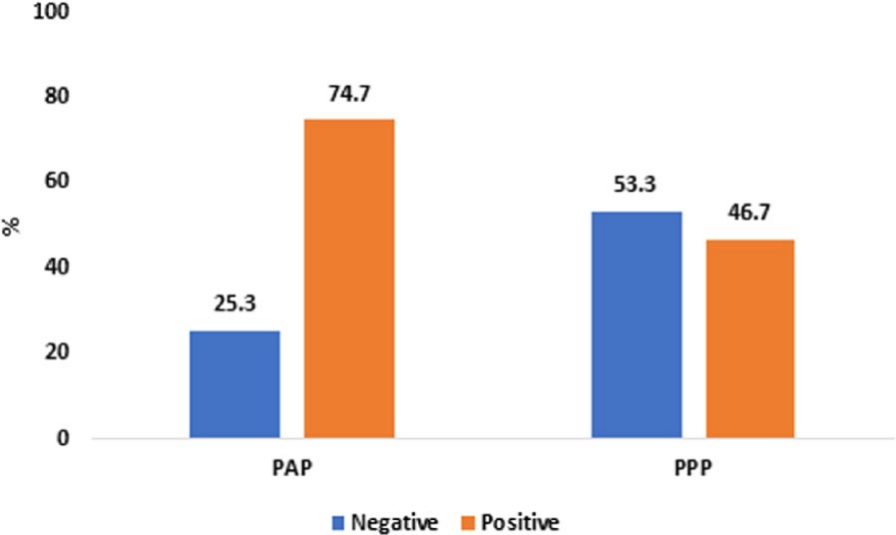
Children’s overall behavior among the study group after intervention

Regarding the overall behavior after intervention, significantly more children in the test group (74.7%) had positive behavior than in the control group (46.7%), (P < 0.0001) (Fig. 2). The mean FIS score before intervention was 2.16 ± 1.18 for the HIQ group, 2.04 ± 1.12 for the AIQ group, and 3.00 ± 1.34 for the LIQ group.

Table 2 displays the logistic regression model for positive behavior as a dependent variable across the intelligence level and intervention. Intelligence had a significant effect on positive behavior. Children with HIQ had significantly higher odds of positive behavior compared to children with LIQ whereas children with AIQ did not differ significantly from children with LIQ as regards to positive behavior (P = 0.01 and 0.09 respectively). The odds for positive behavior were 4.08-fold significantly higher in the PAP technique compared to PPP technique, (OR 4.08, 95% CI 1.71, 9.76, P = 0.002) after adjustment for age, gender, and fear levels. The model goodness of fit indicated a good overall performance. (R^2^ = 0.601).

**Table 2:** Logistic regression model assessing the effect of children’s intelligence levels and intervention on positive behavior

Variables	Wald X^2^	P value	OR	95% CI for OR	
				Lower	Upper
HIQ vs LIQ	6.89	0.01*	4.08	1.43	11.67
AIQ vs LIQ	2.81	0.09	2.40	0.86	6.68
PAP vs PPP	10.01	0.002*	4.08	1.71	9.74

^*^Statistically significant at P < 0.05

*HIQ* high intelligence quotient group, *AIQ* average intelligence quotient group, *LIQ* low intelligence quotient group, *PAP* parental active presence, *PPP* parental passive presence. Children’s overall behavior was adjusted for age, gender, and fear level. Wald X^2^: Wald Chi square test. *OR* odds ratio, *95% CI* confidence interval

Three logistic regression models assessing the effect of intervention on positive behavior for each intelligence level are shown in Table 3. The odds for positive behavior were 13.17-fold higher in the HIQ group children managed with the PAP technique than children managed with the PPP technique (OR 13.17, 95% CI 1.33, 130.74, *P* = 0.03). Similarly, in the LIQ group, 4.14-fold significantly higher odds were found among the children managed with the PAP technique compared to the children managed with the PPP technique (OR 4.14, 95% CI 1.05, 16.26, *P* = 0.04).The odds of positive behavior in the AIQ children managed with the PAP technique was 1.45 times more than those managed with the PPP technique; however, the difference was not statistically significant. (OR 1.45, 95% CI 0.29, 7.26, *P* = 0.65). All models were adjusted for age, gender, and fear levels. The adjusted R^2^ values were 0.543, 0.521, and 0.503 for HIQ, AIQ, and LIQ models, respectively.

**Table 3:** Logistic regression models assessing the relation between intervention and positive behavior per study group

Groups	Variables	Wald X^2^	P value	OR	95% CI for OR	
					Lower	Upper
HIQ	PAP vs PPP	4.85	0.03*	13.17	1.33	130.74
AIQ	PAP vs PPP	0.21	0.65	1.45	0.29	7.26
LIQ	PAP vs PPP	4.13	0.04*	4.14	1.05	16.26

^*^Statistically significant at P < 0.05. *HIQ* high intelligence quotient group, *AIQ* average intelligence quotient group, *LIQ* low intelligence quotient group, *PAP* parental active presence, *PPP* parental passive presence. Children’s overall behavior was adjusted for age, gender, and fear level. Wald X^2^: Wald Chi square test. *OR* odds ratio, *95% CI* confidence interval

## Discussion

This study was performed to determine the effect of parental active and passive presence techniques on the overall behavior of children with different intelligence levels in the dental clinic. Children with different IQ levels who were managed with the PAP technique showed higher overall positive behavior in the dental setting compared to those managed with the PPP technique. Hence, the null hypothesis was rejected.

In correspondence to previous studies [5, 6, 10], the results of this study showed that HIQ children were significantly cooperative during dental treatment compared to LIQ children. Moreover, the PAP technique significantly reinforced the children's positive behavior compared to the PPP technique. The active parental participation helped in alleviating the anxiety induced by their overactive minds due to negative perceptions and anticipated threats that might occur in the dental environment [32].

In the present study, it was observed that LIQ children showed more negative behavior in the dental setting. They were scared, unsecured, and tried to avoid any contact with the dentist, despite the non-pain-provoking intervention that was carried out to eliminate the pain-induced fear. This could be referred to the LIQ being a constant risk factor for the appearance and continuity of low self-esteem and anti-social behavior in the course of life [33]. This observation goes along with previous studies [5, 8, 9] which reported that LIQ children probably needed significantly longer time to accept the dental treatment, thus expressed negative behavior. However, it contradicts other studies that observed inverse [34] or no correlation [10]. Nevertheless, the application of the PAP technique was shown to influence the positive behavior of LIQ children significantly as the parents helped by explaining the procedure and the dentist's instructions and by reassuring the children with physical contact.

The effect of PAP/PPP techniques on the behavior of AIQ children was not significantly displayed in the current study. This could be attributed to some factors that were proposed to impact the children’s behavior including parents' intelligence quotient [35], parenting styles [36] and anxiety [37], and children's emotional quotient [10]. It could be hypothesized that the behavior of AIQ children was coincidentally influenced by some or a combination of these factors. Accordingly, the effect of the PAP/PPP techniques was hindered.

The present study calls attention to the value of recognizing the children's IQ as a predictor of their behavior. Moreover, it highlights the remarkable effect of active parental participation on the children's positive behavior in the dental environment, hence; assists the pediatric dentist in providing the needed quality dental care.

To our knowledge, this is the first clinical trial that has assessed the effect of the PAP/PPP techniques on the overall behavior of children with adjustment of age, gender, and fear levels. However, the limitation imposed by this trial was not assessing the parenting styles and parental anxiety in relation to the children's behavior in the dental operatory. Consequently, further studies are needed to evaluate the effects of these relationships and to evaluate the effectiveness of the PAP/PPP techniques with more pain-provoking dental procedures.

## Conclusions

The PAP technique had positively influenced the children’s overall behavior with different intelligence levels. High IQ children showed more positive behavior than LIQ children who had shown more dental fear in the dental setting.

## Supplementary Information

**Additional file 1:** CONSORT checklist.

**Additional file 2:** Study protocol.

## Data Availability

The datasets used and/or analyzed during the current study are available from the corresponding author on reasonable request.

## Abbreviations

DFA: Dental fear and anxiety
PAP: Parental active presence
PPP: Parental passive presence
TSD: Tell-show-do
IQ: Intelligence quotient
FIS: Facial image scale
FRBS: Frankl behavior rating scale
HIQ: High intelligence quotient
AIQ: Average intelligence quotient
LIQ: Low intelligence quotient
SB-IV: Stanford Binet intelligence scale, fourth edition
DF: Dental fear
OR: Odds ratio
CI: Confidence interval
