# Supplementary material for: Influence of active versus passive parental presence on the behavior of preschoolers with different intelligence levels in the dental operatory: a randomized controlled clinical trial
Source: BMC Oral Health. 2021 Aug 28;21:420. doi: 10.1186/s12903-021-01781-z (PMC8401033; doi:10.1186/s12903-021-01781-z)
Supplement: Supplementary file 2 — Additional file 2: Study protocol. [file 12903_2021_1781_MOESM2_ESM.pdf]

Influence of active versus passive parental presence on the behavior of preschoolers with different intelligence and fear levels in the dental operatory: a randomized controlled clinical trial

Abstract

Background: Dental fear and anxiety still pose the most common factors proposed for the child's negative behavior in the dental operatory. Intelligence has an impact on the children's communication, feelings, and responsiveness to dental situations. The benefits of parental presence on reinforcing the child's behavior during dental treatment is still debatable. **Purpose of the Study:** The aim of this study is to assess the effect of parental active versus parental passive presence (PAP/PPP) technique on the overall behavior of preschool children with different intelligence and fear levels. **Methods:** This study will be two-parallel arms randomized controlled clinical trial. One hundred and fifty healthy children, 3-6-year-old, with no history of previous dental pain/treatment, and intelligence quotient (IQ) level of 70 - ≤110 will be recruited from the outpatient clinic of the Pediatric Dentistry Department, Faculty of Dentistry, Alexandria University, Egypt. In the first visit, the participants will be stratified into 3 equal groups (high, average, low) IQ using the Arabic version of Stanford Binet intelligence scale, fourth edition (SB- IV). In the second visit, dental fear will be assessed using facial image scale (FIS) followed by randomly dividing each IQ group into test and control sub-groups. During intervention, the control sub-groups will be managed using PPP technique, while the test sub-groups will be managed using PAP technique. The dental session will be video recorded and the overall behavior during treatment will be assessed using Frankl behavior rating scale (FBRS) at the end of the second visit.

Clinical relevance: This study aims to bridge the gap between the children's social/ emotional development and their cognitive development, which has an impact on their behavior in the dental clinic. Moreover, it will also highlight the suitable parental role during treatment whether actively participating or only passively present with his child.

Keywords: Behavior modification, Dental fear, Intelligence quotient, Parental active/passive presence, Preschool children, Stanford Binet intelligence scale

Introduction & Literature review

Many practicing dentists have considered the fearful uncooperative child patient as the most troublesome problem in their clinical work. [1] Fear is a reaction to real or imagined threat and is considered as an integral and adaptive aspect of normal development. [2] It is a common occurrence characterized by an essential and inevitable emotion that appears as a response to various dental procedures. [3]

Dental fear has been constantly noticed among children younger than three years, yet it also appears in older children and adolescents. [3] Various measures have been developed in a bid to develop a uniform method of assessing and grading dental fear in children. Examples of these measures include the Children's Fear Survey Schedule (CFSS) developed by Scherer and Nakamura, [4] and Dental Subscale of Children's Fear Survey Schedule (CFSS-DS) developed by Cuthbert and Melamed. [5] These include a questionnaire filled by the child or his parent. [6]A degree of bias has been reported due to the doubt that the child could not fill out a questionnaire. So various techniques have been developed to circumvent this problem.

Picture scales allow for limited cognitive and linguistic skills. They can be easily administered and scored in a clinical context. [6] The most developed picture tests are the Facial Image Scale (FIS), and Children's Dental Fear Picture test (CDFP). [7, 8] The Facial Image Scale uses faces as an indicator of fear. It is also suitable for young preliterate children. [9] The tool has found to show a high correlation with the Venham Picture Test (VPT) when tested for validity. [7, 10]

Another worth discussing issue is the child intelligence, which might affect his behavior in the dental clinic. According to Piaget, intelligence is the ability to adapt to the environment. Thought does not develop as do height and weight, which only increase in magnitude with age. Thought assumes qualitatively different patterns at succeeding age levels. [12]

The term cognition refers to the highest levels of various mental processes such as perception, memory, abstract thinking, reasoning, and problem solving as well as the more integrative and control processes related to executive functions such as planning, choosing strategies, and the enactment of these strategies. [13] Individuals differ from one another in their ability to understand complex ideas, and to adapt effectively to the

environment. [14] Intelligence was further defined according to two themes: the individual's learning from experience, and the individual's ability to adapt to the environment. [15]

Tests of intelligence come in many forms. Some use only a single type of item or question, such as the Peabody Picture Vocabulary Test (PPVT), and Raven's Progressive Matrices. The more familiar measures of general intelligence-such as the Wechsler Tests and the Stanford - Binet Test include many different types of items, both verbal and nonverbal. [16]

The Stanford – Binet Test (Fourth edition) is based on a hierarchical model of intelligence. The four main areas assessed are Verbal reasoning, Abstract/Visual reasoning, Quantitative reasoning, and Short-term Memory. [17] Fifteen subtests are partitioned in to four main reasoning. [18] The Stanford-Binet IV is a useful test in assessment of a broad range of intellectual abilities. [19] The full battery test is not necessarily used to measure the intelligent Quotient (IQ), an item-reduction short form proved to be a more comparable estimate of the full battery composite. [20]

Simpson and others (2002) evaluated intellectual giftedness in 20 gifted children and 20 non-gifted children. They examined the extent of the difference in IQ scores obtained on the two tests and whether order effects were present using The Wechsler Intelligence Scale for Children–Third Edition (WISC-III) and the Stanford-Binet Intelligence Scale 4th Edition (SB-IV). Results showed that the SB-IV Composite Score was significantly higher than the WISC-III Full Scale IQ for both groups. [21]

A study of children's individual characteristics including age, sex, intelligence quotient and personality variables may help in the understanding of their dental fear. [22] Rud and Kisling (1973) investigated the influence of mental development on children acceptance of dental treatment, by using Banit's, Cattells's, and Leiter's methods on 108 individuals with age of (3-9 years). They reported that children with lower IQs showed more fearful behavior. [23] Savin, and Maxim (2008) carried out a study on 88 subjects (54 girls and 34 boys) aged between 6-8 years old: They divided them into 64 normal subjects from psycho-mental viewpoint and 24 subjects with audio sensory disabilities. They made a complex assortment of investigations: projective draw test (thematic projective test), Raven's progressive matrix test and a questionnaire. The results of all the tests showed that there was a significant correlation between the

IQ level and the behavioral conduct manifested by the child. The subjects with higher IQ level presented a normal conduct. [24]

Communicative management is an ongoing subjective process that becomes an extension of the personality of the dentist. Associated with this process are the specific techniques of Tell Show-Do technique, Parental presence/absence, [25] and others. [9] Parents exert a significant influence on their child’s behavior, especially if they have had previous negative dental experiences. [26, 27] The presence or absence of the parent sometimes can be used to gain cooperation for treatment. [9] Parents' insistence to be present during their child’s treatment does not mean they intellectually distrust the dentist. It might mean they are uncomfortable if they visually cannot verify their child’s safety. Therefore, parental presence should not cause any conflict within the dentist. On the other hand, parental presence could be used actively or passively to during dental treatment. [28]

It is evident that a strong relationship exists between dental fear and children’s behavior. [3, 6, 29, 30] However, limited research has dealt with the effect of children's intelligence on their fear and consequently on their behavior in the dental clinic. [31] In addition, no research has investigated active versus passive presence of parents in the dental operatory and its effect on the child’s behavior. These problems have furnished the stimulus for the present investigation.

Hypothesis:

Null Hypothesis: there is no effect of Parental Active/ Passive Presence Technique and Tell Sow Do (TSD) Technique on the behavior of preschool children with different levels of intelligence and fear.

Aim of the study

This study will be conducted to investigate the effect of parental active/ passive presence technique on the overall behavior of preschool children with different levels of intelligence and fear.

Material and Methods

Study Design

This parallel design randomized controlled clinical trial will be set according to the CONSORT statement. [32] The PICO question is: In preschool children with different fear and intelligence levels (P) how does parental active presence technique (I) compared to parental passive presence technique (C) affect the children over all behavior during preventive treatment (O)?

Study Setting

The children will be recruited from the Outpatient clinic of Pediatric Dentistry in Faculty of Dentistry at Alexandria University, Egypt

Study Sample

Sample size estimation

Sample size was based on assuming 5% alpha error, 20% beta error, allocation ratio between test and control subgroups of 1:1, and probability of positive behavior in the control subgroup of low intelligence quotient (IQ) = 0.25. [33] An estimation was made of the probability of positive behavior in the test subgroup with low IQ to have positive behavior as healthy children = 0.87. [34] So, to detect the difference between control and test groups, it was calculated that 9 children per subgroup would be needed. [35] To ensure adequate power, we estimated a reduction in the difference between test and control groups regarding probabilities of positive behavior of 50% with calculated required number= 24. This was increased to 25 children per subgroup to account for non-completion. Thus, the total required number of children= number of groups X number of subgroups X number per group= 3 X 2 X 25= 150 children.

Visual Screening and parent meeting will be carried out to identify children who will fulfill the inclusion criteria.

Inclusion Criteria

1. Age ranging from 3-6 years (the preoperational stage).
2. Patients with at least one sound quadrant.
3. Patients with no history of previous dental treatment and no history of pain
4. Patients with no medical, psychological or mental problems

Exclusion criteria:

1. Multiple dental problems with pain
2. History of previous dental therapy
3. Medical problem
4. Any degree of mental retardation

Ethical Considerations

Ethical approval will be obtained from the Research Ethics Committee, Faculty of Dentistry, Alexandria University before starting the study. A signed informed consent will be obtained prior to treatment (Appendix I) [36] after explaining the objectives, risks and benefits of the study to the children's parents/ guardians and after confirming that all provided personal information will be kept strictly confidential.

Parents and children will be provided age-appropriate dental health education and the proper oral hygiene measures including proper brushing twice a day especially before bedtime and proper flossing if indicated will be demonstrated on a model. A fluoridated toothpaste and a brush will be provided to each participant on the day of recruitment.

If the child has any decayed tooth indicated for restoration, it will be restored in visits other than the study visits. All the possible clinical and/ or adverse outcomes will be explained to parents and they will be asked to report immediately if any of them occurs.

Randomization and allocation concealment

Children will be stratified based on their IQ into 3 groups: high, average, and low IQ groups. In each group, children will be randomly and equally allocated according to intervention into test and control sub-groups. The study will thus include

6 subgroups. Randomization will be performed by a trial independent person using computer random number generator. Randomization sequence in blocks of 2 will be created using random allocation software version 1.0.0. [37] The allocated group will be written on piece of paper which will be folded and enclosed in a sealed envelope carrying the child's name on its cover. At the time of intervention, an assistant will open the envelope, identify the sub-group to which the child will be assigned.

Calibration and reliability of examiner

Prior to the study, the researcher will be calibrated by conducting training sessions with the supervisor for the application of the three measurement scales. Intra-examiner reliability will be assessed by the application of Facial Image Scale (FIS) and Frankl's Behavior Rating Scale (FBRS) to 10 children who will not participate in the study then they will be re-evaluated after 5 days. Then the results will be assessed using Kappa statistics.

Intervention

This study will comprise two visits. (Figure 1).

First visit

Children who will fulfill the inclusion criteria will be evaluated then divided into 3 equal groups according to their level of intelligence IQ by means of Stanford Binet Intelligence Scales, Fourth Edition (SB: IV) - Arabic version [38,39] (Appendix II) after special training.[1] It is a standardized test that measures intelligence and cognitive abilities in children and adults, from age two through mature adulthood. [38]

The test is grouped into four area scores. The four main areas to be assessed are verbal reasoning, abstract/visual reasoning, quantitative reasoning, and short-term memory. There are 8 subtests selected from the total subtest of Stanford Binet Intelligence Scales according to the age group. The reasoning and the subtests links to [38]:

I- Verbal reasoning (VR):

1. Vocabulary (V)

[1] Stanford Binet Intelligence Scales, Fourth Edition - Arabic version: Assessment Course, Steps Training Center, Alexandria. Egypt

2. Comprehension (Com)
3. Absurdities (Ab)

II- Abstract/visual reasoning (A/VR):

1. Pattern (P)
2. Copy (Cop)

III- Quantitative reasoning (QR):

1. Quantitative (Q)

IV- Short-term memory (STMR):

1. Bead memory (BM)
2. Memory for sentence (MS)

Verbal reasoning (VR):

The Verbal Reasoning area score measures verbal knowledge and understanding obtained from the school and home learning environment and reflects the ability to apply verbal skills to new situations. Examples of subtests comprising these factor measure skills which include: word knowledge (Vocabulary), social judgment and awareness (Comprehension), and ability to isolate the inappropriate feature in visual material and social intelligence (Absurdities). [38]

Abstract/visual reasoning (A/VR):

The Abstract/Visual Reasoning area score examines the ability to interpret and perform mathematic operations, the ability to visualize patterns, visual/motor skills, and problem-solving skills through the use of reasoning. An example of a subtest which determines the Abstract/Visual Reasoning score is a timed test that involves tasks such as completing a basic puzzle and replicating black and white cube designs (Pattern and Copy). [38]

Quantitative reasoning (QR):

The Quantitative Reasoning area score measures: numerical reasoning (Quantitative). [38]

Short-term memory (STMR):

The Short-Term Memory score measures concentration skills, short-term memory, and sequencing skills. Subtests comprising this area score measure visual short-term memory and auditory short term memory involving sentences sequences (Memory for sentence). In one subtest that measures visual short-term memory, the participant is presented with pictures of a bead design and asked to replicate it from memory (Bead memory). [38]

Test administration

Administration of the Stanford-Binet Intelligence Scale will take between 45 to 90 minutes (including full battery test). Each selected child will be seated in a private quiet room. The Stanford Binet Intelligence Scale will be applied and explained step by step starting from the first reasoning and its items until the last one.

The first step in this test is to apply the first subtest which is the vocabulary (v) to estimate the ***entry level*** of the examined child. The child chronological age will be matched with a suitable paragraph (represents a vocabulary photo) according to a placement test's paper in a vocabulary subtest (V). If the child is successful in the paragraph, he will be moved to the next one to finish this level (each level contains 2 paragraphs). This will be considered the ***basal level***. [40]

The child will be moved from one level to another until he fails in 5 from 6 paragraphs (fail in 3 consequent levels), which represent the ***ceiling level*** (cut-off point). The level with right answers in both paragraphs will represent the entry level. It will be considered the starting level in all tests for each subtest. [40]

The test will stop at the ceiling level. Calculation will be counted by subtracting all wrong answers from the number of the last paragraph. A score will be given for each subtest (Raw Scores). [40]

From the standardized tables; [41] after finishing all items, the raw Score (RS) will be changed to Standard Age Score (SAS) by using first type of table. These Scores for each subtest will be collected and change to Reasoning Standardized Score(R-S-S) by using another table. Finally, by using the last type of tables the score will be changed to Compound Score (C-S) which represents the IQ of the examined child.

Children will be divided into 3 groups according to their level of intelligence IQ as follows:

1. High IQ Children (HIQ): with score of (above 110)
2. Average IQ Children (AIQ): with score of (90-110)
3. Low IQ Children (LIQ): with score of (70-89)

Age-appropriate oral hygiene instructions will be given to the children and their parents at the end of the first visit.

Second visit

Fear will be measured followed by random allocation into test and control sub-groups and implementation of the intervention.

This visit will start by application of Facial Image Scale (FIS) with every child in each group. It is a visual analog scale comprising of a row of five faces ranging from very happy to very unhappy. Children will be asked to point at which face they feel most like at the moment. The face will be scored by giving a value of one to the most positive affect face and five to the most negative affect face with faces 4 and 5 indicating high dental fear (Appendix III). [7]

After that, all procedures will be explained to the child using the *Tell Show Do* (TSD) Technique. [42] Standardized words as well as procedures and environment will be used for each child during intervention in the dental clinic. Children in the control sub-group will be accompanied by their parent who will sit in passively in the dental operatory behind the patient with no eye contact. Children in test sub-group will be accompanied by their parent who will stand in close proximity to their child with hand holding and may help in explaining the dentist's instructions. [27]

Non-pain provoking dental treatment will follow including: oral prophylaxis, fissure sealant [2] and topical fluoride applications. [3]

Outcome assessment

The intervention will be video recorded, and the child's overall behavior in each test and control sub-group will be evaluated using FBRS[26] at the end of the second

[2] bioseal ® Pit and Fissure Sealant,Biodinamica, Madrid. Spain
[3] Sorbet ® Fluoride gel , Keystone Industries, Hollywood Avenue, Cherry Hill.USA

visit by a blinded examiner (Appendix IV). Rating 1 (– –) will be given to the most negative child behavior and rating 4 (++) will be given to the most positive child behavior.

The face will be scored by giving a value of (1) to the most positive effect face and (5) to the most negative face. Faces with values (1,2) will indicate low dental fear, value (3) will indicate moderate dental fear, and faces with values (4,5) will indicate high dental fear. [(7)]

Statistical Analysis

Descriptive statistics will be calculated as frequencies and percent. The comparison between test and control sub-groups will be done using Chi-squared test. Logistic regression analysis will be conducted to assess the effect of the groups (based on IQ level), subgroups (test or control) and confounders (fear, gender, and age) on the outcome (behavior dichotomies into positive and negative behaviors). Statistical analysis will be done using SPSS version 17.0 (SPSS Inc., Chicago, Ill., USA). Significance level will be set at 5 %.

Funding

There will be no funding to support this study.

Problems anticipated

1- Difficulties in recruiting equal number of participants in each IQ group.

2- Drop out of participants in the second visit.

Estimated duration: 12 months.

Tasks	Months											
**Beginning- end of study**	**1**	**2**	**3**	**4**	**5**	**6**	**7**	**8**	**9**	**10**	**11**	**12**
**Proposal writing**	X											
**Training course**		X										
**Purchase of preventive materials**		X										
**Subjects' recruitment**		X	X	X	X	X	X					
**Intervention application**								X	X	X		
**Data management & statistical analysis**											X	X

Estimated budget

Items	Total Price (L.E.)
SB-IV Training course	3000
SB-IV Arabic version	1000
Toothpastes and brushes	3000
Typodont	500
Fissure Sealant	4500
Fluoride	5250
Prophylaxis pastes capsules	1250
Disposable diagnostic kits	1500
others	2000
Video Camera	5000
Statistical Analysis	1000
Computer Services	2000
Printing Services	5000
**Total**	35,000

Publication policy

This research work will be submitted to international journals for publication.

Order of names and correspondence

1- Thiyezen Abdullah Al Dhelai

2- Amani Mohamed Khalil

3- Yasmine Elhamouly

4- Karin ML Dowidar (corresponding author)

Acknowledgments in publication

1- Prof. Maha El Tantawi, Professor of Dental Public Health, Department of Pediatric Dentistry and Dental Public Health, Faculty of Dentistry, Alexandria University, Egypt.

2- Prof. Mahmoud Abdel Halim Mansy, Department of Educational Psychology, Faculty of Education, Alexandria University, Egypt.

References

1- Ingersoll BD. Distraction and Contingent reinforcement with paediatric dental patients. J Dent Child 1984; 51: 203-207.

2- King NJ, Hamilton DI, Ollendick TH. *Children's phobias: A Behavioral Perspectives.* Chichester. Wiley; 1988.

3- Chapman HR, Kirby NC. Dental fear in children: a proposed model. Brit Dent J 1999; 187: 408-412.

4- Scherer MW, Nakamura NY. A fear schedule for children: a factor analytic comparison with manifest anxiety. Behav Res & Therapy 1968; 6:173-182.

5- Cuthbert ML, Melamed BG. A screening device: Children at risk for dental fear and management problems J Dent Child 1982; 49:432- 436.
6- Folayan MO, Kolawole KA. A critical appraisal of the use of tools for assessing dental fear in children. Afri J Oral Health 2004; 1: 54-63.
7- Buchanan H, Niven N: Validation of a Facial Image Scale to assess child dental anxiety. Int J Ped Dent 2002; 12:47-52.
8- Klingberg G, Vamias Lofqvist L, Hwang CF. Validity of the Children's Dental Fear Picture test (CDFP). Eur J Oral Sci 1995; 103: 55-60.
9- American Academy of Pediatric Dentistry. Guidelines on Behavior Guidance for the Pediatric Dental Patient. 2011 accessed at:http://www.aapd.org/media/policies.asp
10- Buchanan H, Niven N: Further evidence for the validity of the Facial Image Scale. Int J Ped Dent. 2003 13:368-369.
11- Piaget J. *The Language and Thought of the Child.* 3rd Ed. New York: Routledge and Kegan, Ltd; 1959.
12- Sparrow SS, Davis SM. Recent advances in the assessment of intelligence and cognition. J Child Psycho Psychiat 2000; 41: 117-131.
13- Stemberg, RJ, Detterman, DK. *What is Intelligence? Contemporary Viewpoints on its Nature and Definition.* Norwood, NJ: Ablex .1986.
14- Williams W.M. Consequences of how we define and assess intelligence. Psycho Public Policy and Law 1996; 2:506-535.
15- Ulric Neisser et al. Intelligence: Knowns and unknowns. A Psycho 1996; 51:77-101.
16- Roid GH. *Stanford-Binet Intelligence Scales – Examiner's Manual: 5th Ed.* Itasca, IL: Riverside Publishing company. 2003.
17- Kline RB. Is the fourth edited Stanford-Binet a four- factor test? Confirmatory factor analysis of alternative models for ages 2 through 23. J Psycho Assess 1989; 7:4-13.
18- Johnson DL, Howie VM, Owen M, Baldwin CD, Luttman D. Assessment of three-year-olds with the Stanford-Binet Fourth Edition. Psychol Rep 1993; 73:51-7.
19- Nagle RJ, Bell NL. Validation of an item-reduction short form of the Stanford-Binet Intelligence Scale: Fourth Edition with college students. J Clin Psychol 1995 Jan; 51:63-70.

20- Simpson M, Carone DA, Burns WJ, Seidman T, Montgomery D, Sellers A. Assessing giftedness with the WISC-III and the SB-IV. Psychology in the Schools 2002; 39:515-524.
21- Toledano M, Osorio R, Agullera FS Pegalajar J. Children's dental anxiety: influence of personality and intelligence factors M. Int J Ped Dent 1995; 5: 23-28.
22- Rud B, Kisling E. The influence of mental development on children's acceptance of dental treatment. Scand J Dent Res .1973; 81: 343-352.
23- Savin C,Maxim A. The role of the psychological factor in pediatric dental management. J Prev Med 2008; 16: 132-140.
24- Frankl S, Shiere F, Fogels H. Should the parent remain with the child in the dental operatory?.J Dent Child 1962; 29:150-163.
25- Klingberg G, Berggren U. Dental problem behaviors in children of parents with severe dental fear. Swed Dent J 1992;16:27-32, 39.
26- Baier K, Milgrom P, Russell S, Mancl L, Yoshida T. Children's fear and behavior in private pediatric dentistry practices. Ped Dent 2004; 26:316-21.
27- Pinkham JR. An analysis of the phenomenon of increased parental participation during the child's dental experience. J. Dent Child 1991;58:458-63.
28- Venham LL, Murray P, Kremer EG. Personality Factors affecting the preschool child's response to dental stress. J Dent Res 1979; 58:2046-2051.
29- Rãducanu AM et al. Assessment of the prevalence of dental fear and its causes among children and adolescents attending a department of pediatric dentistry in bucharest. OHDMBSC 2009; 8:42-49.
30- Sawtel R.O, Simon, JF, Simonsson RJ. The effect of five preparatory methods upon child behavior during the first dental visit. J Dent Child 1974; 41:37-45.
31- Fields H, Pinkham JR. Videotape Modeling of the Child Dental Patient. JADA 1976; 55:958-963.
32- Schulz KF, Altman DG, Moher D. CONSORT 2010 statement: updated guidelines for reporting parallel group randomised trials. BMC medicine. 2010; 8:18.
33- Fields H, Pinkham J. Videotape Modeling of the Child Dental Patient. J Dent Res. 1976;55:958–963.

34- Peretz B, Gluck G. Magic trick: a behavioural strategy for the management of strong-willed children. Int J Paediatr Dent. 2005;15:429–436.

35- The University of British Columbia:Sample size Calculator for comparing two proportional Means. https://www.stat.ubc.ca/~rollin/statsssize/b2.html. (2017) Accessed 22 October 2017.

36- American Academy of Pediatric Dentistry. Guidelines on informed consent. 2011 accessed at: http://www.aapd.org/media/policies.asp

37- Saghaei M. Random allocation software for parallel group randomized trials. BMC Med Res Methodol. 2004;4:26.

38- Kamel ML. *Stanford-Binet Intelligence Scale- Guide for Administration and Scoring: Fourth Edition.* 2nd Ed. Cairo: Dar Al Nahdah Al Arabia; 1998.

39- Thorndike RL, Hagen E, Sattler J. *Stanford-Binet Intelligence Scale- Guide for Administration and Scoring:* 4th Ed. Chicago: Revised Publishing Co; 1986.

40- Kamel ML. *Stanford-Binet Intelligence Scale- Record Booklet: Fourth Edition.* 2nd Ed. Cairo: Dar Al Nahdah Al Arabia; 1998.

41- Kamel ML. *Stanford-Binet Intelligence Scale- Standardized tables: Fourth Edition.* 2nd Ed. Cairo: Dar Al Nahdah Al Arabia; 1998.

42- Addelston H. Child patient training. Fortnightly Review of the Chicago Dental Society 1959; 38.

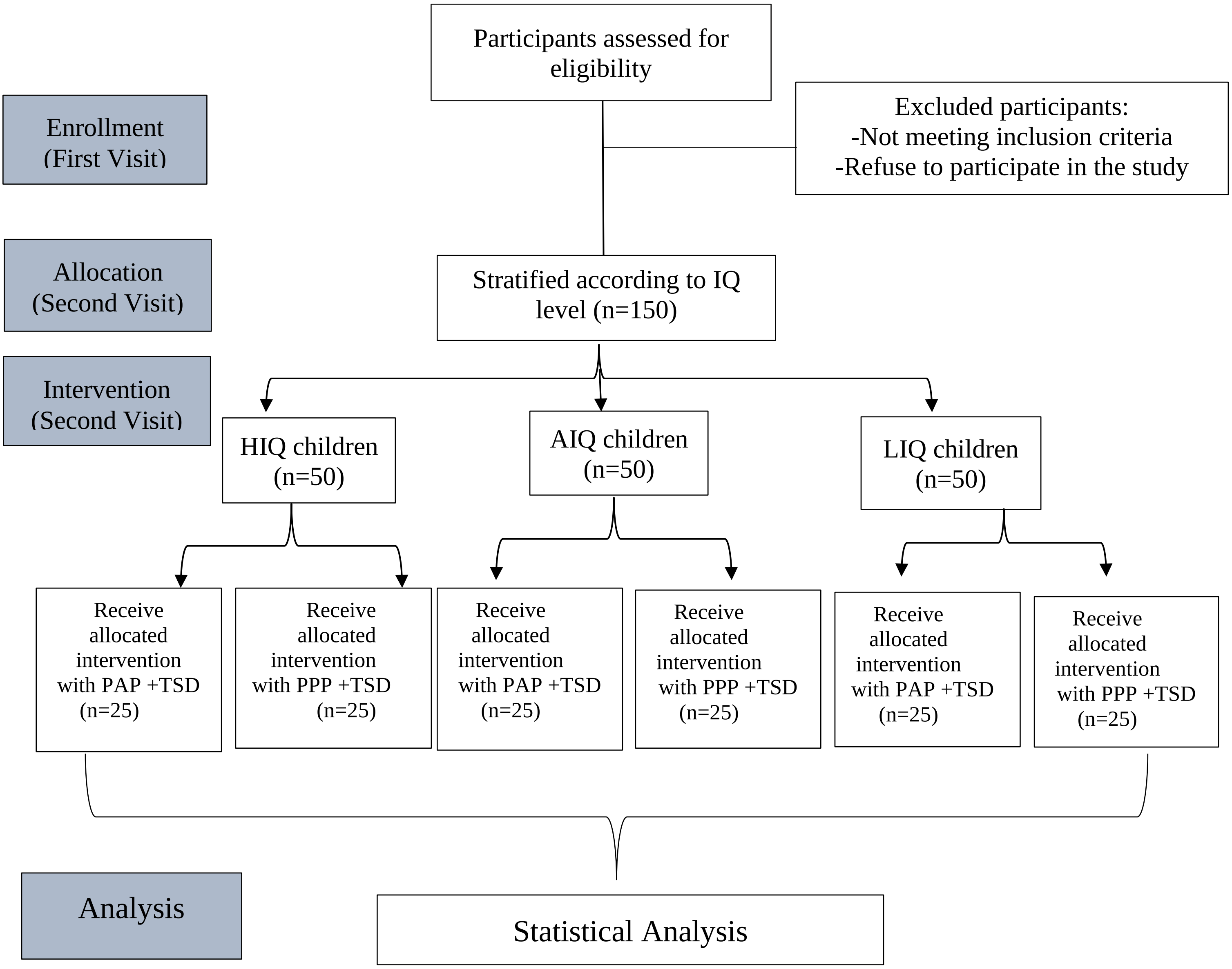

Figure 1: Study Flow diagram

Appendix I

نموذج الموافقة المستنيرة للاشتراك في دراسة علمية

اسم الباحث / الباحثين:` ذي يزن عبد الله احمد
القسم: طب اسنان الاطفال
عنوان الدراسة: تأثير مستوي الذكاء على مدي الخوف عند اطفال ما قبل المدرسة وعلى سلوكهم في عيادة الاسنان باستخدام اسلوب وجود الوالدين الايجابية/السلبية
القسم: طب أسنان أطفال

1- **مقدمة:** ومستوي الذكاء لدي الأطفال و مدى الخوف من طبيب الاسنان من الاسباب التي قد تؤثر على سلوك الطفل وتقبله للعلاج من طبيب الاسنان.

2- **الهدف من الدراسة:** دراسة وتقييم مدي تأثير ومستوي الذكاء لدي الاطفال في مدي خوفهم من طبيب الاسنان وسلوكهم وطبيعة تقبلهم للعلاج لدي طبيب الاسنان

3- عدد الأشخاص المشاركين: 150 شخص
4- طريقة الدراسة: سوف يتم اجراء فحص لمستوي الذكاء للأطفال ثم سيتم اجراء الوقاية السنية اللازمة لحماية اسنانهم.
5- فترة **مشاركة طفلي/طفلتي بالدراسة:** حتى انتهاء فترة العلاج
6- مخاطر الدراسة: لا يوجد أي مخاطر
7- فوائد الدراسة: معرفة مدي اهمية ذكاء الطفل في استجابته للعلاج عند طبيب الاسنان
8- سرية المعلومات: سوف يتم حفظ المعلومات المتعلقة بالطفل في سرية تامة ولن يتم التعريف به في أي تقرير أو نشر لنتائج الدراسة
9- وسائل علاج أي اصابة ربما تحدث من الدراسة:
سوف يقوم الباحث باتخاذ جميع الوسائل لمنع أي إصابة ممكن أن تحدث نتيجة هذه الدراسة. ولكن إذا حدثت أي إصابات غير متوقعة نتيجة اشتراكه بالدراسة سوف يتلقى العلاج اللازم بالكلية.
10- تكاليف الاشتراك بالدراسة: لا يوجد.
11- لن تتلقى أي أموال للاشتراك بالدراسة.
12- حقوقك كمشارك في الدراسة:
الاشتراك تطوعياً ولك الحق في أن تستمر أو تنسحب من الدراسة في أي وقت دون تبعات عليك مع توضيح أسباب الانسحاب.
13- للاستفسار عن الدارسة أو عند حدوث أي مشاكل أو شكوى غير متوقعة أو شعرت بأن أشياء غير طبيعية أو معتادة تحدث. يمكنك الاتصال بنائب رئيس لجنة أخلاقيات البحث العلمي للقسم أ.د هناء رسلان
أو مقابلته بقسم: طب اسنان الاطفال   تحويلة: 221   **ت:** 4868066/48696901
14- اقرار الباحث:
لقد قمت بشرح الدراسة بالتفصيل والهدف منها وطريقة الدراسة والمخاطر والفوائد المتعلقة بالدراسة. مع الإجابة عن أي أسئلة آثارها المشارك,, وسوف التزم بخطوات الدراسة كاملة وبالمعايير الأخلاقية والقانونية للبحث العلمي بالكلية.
توقيع الباحث: ____________________ التاريخ:
15- توقيع المشارك:
أنا الموقع أدناه والد الطفل قد شُرح لي الهدف من الدراسة وطريقتها وفهمت الفوائد والمخاطر المتعلقة بالدراسة. وأخذت نسخة من هذه الموافقة وقد تم اعطائي الفرصة للسؤال والاستفسار قبل التوقيع وقيل لي أن من حقي الاستفسار في أي وقت لاحق. وأنا أتطوع للمشاركة في هذه الدراسة ولي الحق في الانسحاب في أي وقت دون تبعات عليّ. وأوافق على التعاون مع الباحث وإخباره مباشرة بأي مشاكل غير متوقعة يمكن أن تحدث أثناء اشتراك ابنى/ابنتى في الدراسة.
- لقد قرأت وفهمت المعلومات المذكورة في هذه الموافقة.
- لقد تم شرح المعلومات المذكورة لي وفهمتها.

اسم وتوقيع المشارك والد الطفل: ________________ التاريخ:
اسم وتوقيع الشاهد:______________________ التاريخ:

توقيع رئيس لجنة اخلاقيات البحث العلمي:__________ التاريخ: الختم

Appendix I

Informed Consent Form to Participate in a Research Study

Alexandria University, Faculty of Dentistry

Name of the researcher: Thiyezen Abdullah Al Dhelai

Department: Department of Pediatric Dentistry and Dental Public Health, Faculty of Dentistry, Alexandria University, Egypt.

Title of the research: Influence of active versus passive parental presence on the behavior of preschoolers with different intelligence and fear levels in the dental operatory: a randomized controlled clinical trial

1. **Introduction:** The children's intelligence level and the extent of fear of the dentist are among the reasons that may affect the child's behavior and his acceptance of treatment by the dentist
2. **Purpose of the research:** Assess the effect of parental active versus parental passive presence technique on the overall behavior of preschool children with different intelligence and fear levels.
3. **Number of participants:** 150
4. **Study type:** clinical trial
5. **Expected Duration:** 12 months
6. **Risks:** There are no expected health risks.
7. **Benefits:** Your participation is likely to help us correlate the children's social/ emotional development and their cognitive development, which might influence their behavior in the dental setting as well as identifying the appropriate parental role during treatment.
8. **Confidentiality:** The information that we collect from this research project will be kept private. Any information about you will have a number on it instead of your name.
9. **Methods of treating any injury that may occur from the study:** The researcher will take all measures to prevent any injury or disease that may occur as a result of this study. But if any unexpected injury occurred as a result of your participation in the study, you will receive the necessary treatment at the college.
10. **Reimbursements:** You will not be provided any incentive to take part in the research
11. **You will not receive any money to participate in the study**
12. **Right to refuse or withdraw:** You do not have to take part in this research if you do not wish to do so. You may stop participating in the study at any time that you wish.

13. **If you have any questions**, you can ask them now or later. If you wish to ask questions later, you may contact: [Thiyezen Abdullah Al Dhelai: 010083676202] or report any problem to Prof. Hanaa Raslan, ethics committee coordinator: 03486969/4868066 Ext: 221

14. Statement by the researcher/person taking consent

I confirm that the participant was given an opportunity to ask questions about the study, and all the questions asked by the participant have been answered correctly and to the best of my ability. I confirm that the individual has not been coerced into giving consent, and the consent has been given freely and voluntarily.

A copy of this ICF has been provided to the participant.

Print Name of Researcher/person taking the consent________________________

Signature of Researcher /person taking the consent __________________Date   Day/month/year

15. Certificate of Consent

I have read the foregoing information, or it has been read to me. I have had the opportunity to ask questions about it and any questions I have been asked have been answered to my satisfaction. I consent voluntarily to be a participant in this study

Print Name of Participant__________________

Signature of Participant ___________________Date _________________   Day/month/year

Appendix II

Stanford Binet Intelligence Scale 4th Ed (SB-IV)

Arabic Version

Child Name: **Age:** **Gender:**

Address: **Phone No.:**

EL:

IQ Calculation from standard tables							
MR	S	RS	SAS	Total	R-S-S	Total	C-S = IQ
VR	V						
	Com						
	Ab						
A/VR	P						
	Cop						
QR	Q						
STMR	BM						
	MS						
Note:							

Abbreviations	
EL	Entry Level
MR	Mean Reasoning
S	Subtests
RS	Raw Score
SAS	Standard Age Score
R-S-S	Reasoning Standardized Score
C-S	Compound Score
VR	Verbal reasoning
V	Vocabulary
Com	Comprehension
Ab	Absurdities
A/VR	Abstract/visual reasoning
P	Pattern
Cop	Copy
QR	Quantitative reasoning
Q	Quantitative
STMR	Short-term memory
BM	Bead memory
MS	Memory for sentence

Intelligence Quotient IQ Classification Guide	
IQ Range	General Classification
140 and up	Very Superior
120-139	Superior
110-119	High Average
90-109	Average
80-89	Low Average
70-79	Borderline Impaired
Sources; Thorndike, Hagen, and Sattler, 1986	

I- Verbal reasoning (VR)-الاستدلال اللفظي

1- Vocabulary (V) - المفردات					
Record Answers – Draw Circle on(+) or (-)					
Age	Level	V Photos		V	Answer
2	A	+	-	1	
		+	-	2	
3	B	+	-	3	
		+	-	4	
	C	+	-	5	
		+	-	6	
4	D	+	-	7	
		+	-	8	
	E	+	-	9	
		+	-	10	
5	F	+	-	11	
		+	-	12	
6	G	+	-	13	
		+	-	14	
		V Words			
7-8	H	+	-	15	
		+	-	16	
9	I	+	-	17	
		+	-	18	
10-11	J	+	-	19	
		+	-	20	
12-13	K	+	-	21	
		+	-	22	
14-15	L	+	-	23	
		+	-	24	
16 +	M	+	-	25	
		+	-	26	
	N	+	-	27	
		+	-	28	
	O	+	-	29	
		+	-	30	
	P	+	-	31	
		+	-	32	
	Q	+	-	33	
		+	-	34	
	R	+	-	35	
		+	-	36	
	S	+	-	37	
		+	-	38	
	T	+	-	39	
		+	-	40	
	U	+	-	41	

		+	-	42	
	V	+	-	43	
		+	-	44	
	W	+	-	45	
		+	-	46	
**(+): Pass**			**(-): Fail**		

**Calculation of Raw Score RS**	
**A-Highest Paragraph Score**	
**B- Total No. of (-)**	
**Raw Score RS (A- B)**	

I- Verbal reasoning (VR)-الاستدلال اللفظي

2- Comprehension (Com) - الفهم					
Record Answers – Draw Circle on(+) or (-)					
Level	Photo		COM		Answer
A	+	-	1	انف	
	+	-	2	فم	
B	+	-	3	شعر	
	+	-	4	رجل	
C	+	-	5	ابهام/ اظفر	
	+	-	6	ذقن/ اظفر	
	Q				
D	+	-	7	جائع	
	+	-	8	عطشان	
E	+	-	9	شراء الاكل	
	+	-	10	الشارع	
F	+	-	11	حمام	
	+	-	12	مظلة	
G	+	-	13	تمشيط شعر	
	+	-	14	حقنة	
H	+	-	15	باب	
	+	-	16	قمامة	
I	+	-	17	بحر	
	+	-	18	اذن	
J	+	-	19	قراءة جريدة	
	+	-	20	حاجة للفلاحين	

K	+	-	21	علامات مرور	
*	+	-	22	تفضيل سيارة	*
L	+	-	23	مجانية تعليم	
	+	-	24	تنقية مياة المجاري	
M*	+	-	25	لمبة كهربائية	*
	+	-	26	تدريبات علي الحريق	
N	+	-	27	الاطباء	
*	+	-	28	التلفون	*
O	+	-	29	لا يعرف الهجاء	
	+	-	30	قروض	
P	+	-	31	اكثر من جريدة	
	+	-	32	ملابس فاتحة	
Q	+	-	33	بيوت المدن	
*	+	-	34	العمل الحر	*
R	+	-	35	مجلس ادارة	
	+	-	36	رخصة قيادة	
S*	+	-	37	سيارة صغيرة	*
	+	-	38	مدة محدودة	
T	+	-	39	حقوق الانسان	
	+	-	40	تغيير القوانين	
U	+	-	41	براءة اختراع	
*	+	-	42	اعلانات	*
**(+): Pass**			**(-): Fail**		
***: mean it need 2 answers**					

**Calculation of Raw Score RS**	
**A- Highest Paragraph Score**	
**B- Total No. of (-)**	
**Raw Score RS (A-B)**	

I- Verbal reasoning (VR)-الاستدلال اللفظي

3- Absurdities (Ab) – السخافا ت					
Draw Circle on Choices (a-c) A-B - Draw Circle on (+) or (-) – S1 Not count					
Level	3 Pic		Ab		Response
A-B			S1	شجرة مقلوبة	a b c
A	+	-	1	سكين منحني	a b c
	+	-	2	قبعة مقلوبة	a b c
B	+	-	3	ولد يمشط شعرة بالملعقة	a b c
	+	-	4	بطة لها اذنا ارنب	a b c
	EXP				
C	+	-	5	القرأة معصوب العينين	
	+	-	6	الكتابة بالشوكة	
D	+	-	7	الكنس	
	+	-	8	الشرب من بزازة طفل	
E	+	-	9	عجلات مربعة الشكل	
	+	-	10	المشي في المطر	
F	+	-	11	سمكة تمشي	
	+	-	12	قطة في قفص	
G	+	-	13	اكل الشوربة بالسكينة	
	+	-	14	تمشيط شعر رأس اصلع	
H	+	-	15	لبس البنطلون مقلوب	
	+	-	16	ركوب البسكليتة في الماء	
I	+	-	17	منشار مقلوب	
	+	-	18	رجلين جالسان في المطر	
J	+	-	19	القفز فوق المنزل	
	+	-	20	الكتابة العكسية	
K	+	-	21	ديك يرقد علي البيض	
	+	-	22	اوزان غير متساوية	
L	+	-	23	طابع البريد في المكان الخطأ	
	+	-	24	ظلال في جهات مختلفة	

M	+	-	25	الارنب داخل في الكلب	
	+	-	26	لا يوجد يوم جمعة	
N	+	-	27	يصوب بندقية	
	+	-	28	الفقاقيع نازلة لتحت	
O	+	-	29	رياح في مختلف الجهات	
	+	-	30	الابهام في الجانب الخطأ	
P	+	-	31	نقطة الارتكاز في الموقع الخطأ	
	+	-	32	امريكا الشمالية والجنوبية	
(+): Pass				(-): Fail	

Calculation of Raw Score RS	
A- Highest Paragraph Score	
B- Total No. of (-)	
Raw Score RS (A-B)	

II- Abstract/visual reasoning (A/VR)-الاستدلال المجرد البصري

4- Pattern (P) – تحليل النمط								
Draw Circle on (+) or (-)or (T-) or (R-) – S 1,2,3,4,5 Not counts								
Level			Degree		Time	P	Response	Cube No.
A	+	-	T-	R-	∞	1	Demo	/
	+	-	T-	R-	∞	2	//	/
B	+	-	T-	R-	∞	3	//	/
	+	-	T-	R-	∞	4	//	/
C	+	-	T-	R-	∞	5	//	/
	+	-	T-	R-	∞	6	//	/
D-E					∞	S1	Cube Demo	1
D	+	-	T-	R-	30 S	7	//	1
	+	-	T-	R-	30 S	8	//	1
E	+	-	T-	R-	30 S	9	//	1
	+	-	T-	R-	30 S	10	//	1
F-I					∞	S2	//	2

**F**	**+**	**-**	**T-**	**R-**	**30 S**	**11**	**//**	**2**
	**+**	**-**	**T-**	**R-**	**30 S**	**12**	**//**	**2**
**G**	**+**	**-**	**T-**	**R-**	**30 S**	**13**	**//**	**2**
	**+**	**-**	**T-**	**R-**	**30 S**	**14**	**//**	**2**
**H**	**+**	**-**	**T-**	**R-**	**30 S**	**15**	**//**	**2**
	**+**	**-**	**T-**	**R-**	**30 S**	**16**	**//**	**2**
**I**	**+**	**-**	**T-**	**R-**	**30 S**	**17**	**//**	**3**
	**+**	**-**	**T-**	**R-**	**30 S**	**18**	**//**	**3**
**J-L**					∞	**S3**	**Cube Demo**	**4**
**J**	**+**	**-**	**T-**	**R-**	**45 S**	**19**	**//**	**4**
	**+**	**-**	**T-**	**R-**	**45 S**	**20**	**//**	**4**
**K**	**+**	**-**	**T-**	**R-**	**45 S**	**21**	**//**	**4**
	**+**	**-**	**T-**	**R-**	**45 S**	**22**	**//**	**4**
**L**	**+**	**-**	**T-**	**R-**	**45 S**	**23**	**//**	**4**
	**+**	**-**	**T-**	**R-**	**45 S**	**24**	**//**	**4**
**M-O**					∞	**S4**	**Photo only**	**3**
**M**	**+**	**-**	**T-**	**R-**	**30 S**	**25**	**//**	**3**
	**+**	**-**	**T-**	**R-**	**30 S**	**26**	**//**	**3**
**N**	**+**	**-**	**T-**	**R-**	**30 S**	**27**	**//**	**3**
	**+**	**-**	**T-**	**R-**	**30 S**	**28**	**//**	**3**
**O**	**+**	**-**	**T-**	**R-**	**30 S**	**29**	**//**	**3**
	**+**	**-**	**T-**	**R-**	**30 S**	**30**	**//**	**3**
**P-Q**			**T-**	**R-**	∞	**S5**	**//**	**4**
**P**	**+**	**-**	**T-**	**R-**	**45 S**	**31**	**//**	**4**
	**+**	**-**	**T-**	**R-**	**45 S**	**32**	**//**	**4**
**Q**	**+**	**-**	**T-**	**R-**	**45 S**	**33**	**//**	**4**
	**+**	**-**	**T-**	**R-**	**45 S**	**34**	**//**	**4**
**R**	**+**	**-**	**T-**	**R-**	**45 S**	**35**	**//**	**4**
	**+**	**-**	**T-**	**R-**	**45 S**	**36**	**//**	**4**
**S**	**+**	**-**	**T-**	**R-**	**60 S**	**37**	**//**	**6**
	**+**	**-**	**T-**	**R-**	**60 S**	**38**	**//**	**6**

**T**	**+**	**-**	**T-**	**R-**	**90 S**	**39**	**//**	**9**
	**+**	**-**	**T-**	**R-**	**90 S**	**40**	**//**	**9**
**U**	**+**	**-**	**T-**	**R-**	**90 S**	**41**	**//**	**9**
	**+**	**-**	**T-**	**R-**	**90 S**	**42**	**//**	**9**

**(+): Pass**	**(-): Fail**
**(T-): Time Failure**	**(R-): Reversal Failure**

**Calculation of Raw Score RS**	
**A- Highest Paragraph Score**	
**B- Total No. of (-),(T-),(R-)**	
**Raw Score RS (A-B)**	

II- Abstract/visual reasoning (A/VR)- الاستدلال البصري المجرد

**5- Copy (Cop) – النسخ**				
**Draw Circle on (+) or (-) – Starting from G use white papers for drawing**				
**Level**	**Cubes**		**Cop**	**Response**
	**3-4 Cubes need for both of you (Green color)**			
**A**	**+**	**-**	**1**	
	**+**	**-**	**2**	
**B**	**+**	**-**	**3**	
	**+**	**-**	**4**	
**C**	**+**	**-**	**5**	
	**+**	**-**	**6**	
**D**	**+**	**-**	**7**	
	**+**	**-**	**8**	
**E**	**+**	**-**	**9**	
	**+**	**-**	**10**	
**F**	**+**	**-**	**11**	
	**+**	**-**	**12**	
**G**	**+**	**-**	**13**	
	**+**	**-**	**14**	
	**Draw Object**			
**H**	**+**	**-**	**15**	
	**+**	**-**	**16**	
**I**	**+**	**-**	**17**	
	**+**	**-**	**18**	
**J**	**+**	**-**	**19**	
	**+**	**-**	**20**	
**K**	**+**	**-**	**21**	

	+	-	22	
L	+	-	23	
	+	-	24	
M	+	-	25	
	+	-	26	
N	+	-	27	
	+	-	28	
(+): Pass			(-): Fail	

Calculation of Raw Score RS	
A- Highest Paragraph Score	
B- Total No. of (-)	
Raw Score RS (A-B)	

III- Quantitative reasoning (QR)- الاستدلال الكمي

6- Quantitative (Q) – الاختبار الكمي					
Record Answers – Draw Circle on(+) or (-)					
Level	Cubes		Q	Wright Answers	Response
	Explain The Cube and Dotes on each side				
A-C	+	-	1	Cube –Upper side with 1 Dote	The same Cube
	+	-	2	Cube –Upper side with 6 Dotes	The same Cube
D	+	-	3	Cube –Upper side with 3Dotes	The same Cube
	+	-	4	Cube- Upper side with 2 Dotes	Count Dotes
E	+	-	5	Cube- Upper side with 5 Dotes	Count Dotes
	+	-	6	3 Cubes- each -Upper side with 1 Dotes	Count Dotes 1+1+1
F	+	-	7	2 Cubes- each -Upper side with 1 ,2 Dotes	The same Cubes
	+	-	8	2 Cubes- each -Upper side with 2,5 Dotes	The same Cubes
G	+	-	9	3 Cubes- each -Upper side with 2,4,3 Dotes	Count Dotes2+4+3
	+	-	10	2 Cubes- each -Upper side with 4 ,6 Dotes	The same Cubes
H	+	-	11	2 Cubes- each -Upper side with 2 ,4 Dotes	Count Dotes 2+4 =Cube Dotes
	+	-	12	4Cubes- each -Upper side with 1,2, 3,4 Dotes	Complete rank= 5,6
	Q				
I	+	-	13	3 Child	

	+	-	14	**4 Pencils**	
J	+	-	15	**6 Cm**	
	+	-	16	**C**	
K	+	-	17	**50قرش**	
	+	-	18	**B**	
L	+	-	19	**B**	
	+	-	20	**[20-ريال2- 1/2 [4- 1/4 [1-1/2,2-1/4 [10 -1/2<br>شلن ;**	
M	+	-	21	**8 Kg**	
	+	-	22	**10 week**	
N	+	-	23	**1-5 or 20%**	
	+	-	24	**-1/4 1,ريال-1/25- 1/4 [2[ريال , 1شلن , 2-1/24- 1/1<br>]5 1/2- ريال] 1-0شلن**	
O	+	-	25	**6**	
	+	-	26	**120 Pounds**	
P	+	-	27	**يملاء اولا الجردل 5 لتر ثم يملاء الثاني 3 لتر من الاول<br>حيفضل 2 لتر في الاول**	
	+	-	28	**4 Lines**	
Q	+	-	29	**تملي 3 لتر في الاول وتفرغة في الجردل الثاني وتكررها<br>مرتين اخر مرة سيتبقي عندها لتر واحد في الجردل الاول**	
	+	-	30	**24**	
R	+	-	31	**Type 2 Chees**	
	+	-	32	**0.4**	
S	+	-	33	**4 Years**	
	+	-	34	**5 Hours**	
T	+	-	35	**175 Km**	
	+	-	36	**275 قرش**	
U	+	-	37	**18 Lines**	
	+	-	38	**900 بلاطة**	
V	+	-	39	**48 Oranges**	
	+	-	40	**50 Minutes**	

(+): Pass **(-): Fail**

Calculation of Raw Score RS	
A- Highest Paragraph Score	
B- Total No. of (-)	
Raw Score RS (A-B)	

IV- Short-term memory (STMR)-الذاكرة قصيرة المدى

7- Bead memory (Bm) – اذاكرة الخرز				
Draw Circle on (+) or (-) a-e, S1,2,3 not counted<br>Explain Beads with different colors and shapes				
Level	Beads only		Cop	Response
	Show bead shape – let him point on the exact photo			
a	+	-		خرزة زرقاء كروية
b	+	-		خرزقة حمراء قرصية
c	+	-		خرزة بيضاء اسطوانية
d	+	-		خرزة زرقاء مخروطية
	Show bead shape hid it – let him point on the exact photo			
A-C	+	-	1	حمراء اسطوانية
	+	-	2	زرقاء قرصية
D	+	-	3	بيضاء كروية
	+	-	4	حمراء كروية
E	+	-	5	بيضاء مخروطية
	+	-	6	حمراء كروية وزرقاء كروية
F	+	-	7	بيضاء كروية وبيضاء مخروطية
	+	-	8	زرقاء مخروطية وزرقاء اسطوانية
G	+	-	9	حمراء قرصية وبيضاء كروية
	+	-	10	زرقاء اسطوانية وحمراء مخروط
	Bead, Stand			
H-K			S1	
H	+	-	11	
	+	-	12	

I	+	-	13	
	+	-	14	
J	+	-	15	
	+	-	16	
K	+	-	17	
	+	-	18	
L-N			S2	
L	+	-	19	
	+	-	20	
M-N	+	-	21	
	+	-	22	
O-Q			S3	
O-P	+	-	23	
	+	-	24	
Q	+	-	25	
	+	-	26	
R	+	-	27	
	+	-	28	
S	+	-	29	
	+	-	30	
T	+	-	31	
	+	-	32	
U	+	-	33	
	+	-	34	
V	+	-	35	
	+	-	36	
W	+	-	37	
	+	-	38	
X	+	-	39	
	+	-	40	
Y	+	-	41	

	+	-	42	
**(+): Pass**			**(-): Fail**	

**Calculation of Raw Score RS**	
**A- Highest Paragraph Score**	
**B- Total No. of (-)**	
**Raw Score RS (A-B)**	

IV- Short-

term memory (STMR)-الذاكرة قصيرة المدى

**8- Memory for sentence (Ms) – ذاكرة الجمل**				
**Draw Circle on (+) or (-) – S1,2,3 not counted**				
**Level**			**Ms**	**Sentence**
**A-F**			**S1**	بنت طويلة
**A**	+	-	**1**	حصان كبير
	+	-	**2**	اشرب اللبن
**B**	+	-	**3**	عاوزك تبص لي
	+	-	**4**	العربيات تجري بسرعة
**C**	+	-	**5**	الاشجار الخضراء كبيرة
	+	-	**6**	انا رحت البيت
**D**	+	-	**7**	عاوزك تروح لغاية الدكان
	+	-	**8**	روح شوف البلياتشو المضحك
**E**	+	-	**9**	سميرة عندها كلب وقطة
	+	-	**10**	التياترو جاي بلدنا بكرة
**F**	+	-	**11**	هاني هو صديق سامي الجديد
	+	-	**12**	سارة تحب البسكليتة الجديدة بتاعتها
**G-I**			**S2**	ممدوح لعب مع الكلب
**G**	+	-	**13**	الولد الصغير الشقي مش ممكن يبطل عياط
	+	-	**14**	الشمس بتخلي النهار دايما دافي ومنور وجميل
**H**	+	-	**15**	جه وقت نوم الطفل الصغير في سريرة
	+	-	**16**	ابراهيم رسم صورة حلوة لامة بمناسبة عيد ميلادها

I	+	-	17	نصر ما كانش عايز يخرج من السينما قبل ما ينتهي الفلم
	+	-	18	عصام من النوع الي ما يحبش يقضي وقت كثير قدام التلفزيون
J-Q			S3	حبل الطيارة الورق الطويل اتقطع مني
J	+	-	19	الناس ما قدرتش تشوف الطيارة النهاردة الصبح علشان الضباب الكثيف في السما
	+	-	20	مديحة ادت اخوها بمناسبة عيد ميلادة قميص علية خطوط حمرا وبيضا
K	+	-	21	وهو بيجري للاتوبيس وقع علي الارض وجا لة كسر في رجلة اليمين
	+	-	22	صحيح البحر باين هادي النهاردة لكن ممكن يكون العوم فية خطر
L	+	-	23	نيفين وهي بتلعب برة رجليها تزحلقت ووقعت وكل هدومها تغطت بالطين
	+	-	24	حصل النهاردة عطل في موتور الطيارة وعشان كدة عملت هبوط اضطراري
M	+	-	25	امبارح هطول المطر كان غزير وعمل بركة في الشارع بيلعب فيها الاولاد
	+	-	26	توقع جيش العدو الهزيمة ولذلك رفع الراية البيضاء عالية اعلانا للاستسلام
N	+	-	27	فريد جري وراء الكلب حول المنزل ولكن لم يتمكن من الامساك بة
	+	-	28	كانت الطيور تحلق ف يالسماء وتغرد حين استيقظ خليل هذا الصباح
O	+	-	29	امطرت السماء هذا الصباح ولذلك حمل التلاميذ المظلات في طريقهم الي المدرسة
	+	-	30	كان الحراس نائمين الليلة الماضية نوما عميقا ولذلك تسلل اللصوص الي المصنع
P	+	-	31	توجة فريق اللاعبين الي الملعب ولكن هطل المطر بغزارة مما ادي الي تأجيل المبارة
	+	-	32	الجو الحار الرطب الذي كان سائدا في معظم اواخر الصيف الطويل جعل الناس يشعرون غالبا بالضيق
Q	+	-	33	بفضل الحكمة والنظرة البعيدة والتخطيط استطاع الاشقاء تدبير امور حياتهم رغم ضيق ذات اليد
	+	-	34	منصو راستطاع اصلاح نافذة الحجرة قبل عودة ابية ولذلك فانة افلت من العقاب
R	+	-	35	اقلعت السفينة مبكرة من الميناء قبل موعدها بساعة وتركت وراءها صلاح يندب حظة السئ
	+	-	36	لم يستطع السائق التحكم ف يسيارتة بفعل الرياح والامطار فهوت السيارة الي قاع النهر

S	+	-	37	كان من اعظم الامور مشقة وهي وحدها في رحلتها الطويلة وفي يوم حار هو التغلب علي الشعور بالملل
	+	-	38	كان الجو شديد الحرارة والجفاف ف يالصيف فوصل الحال في سبتمبر الي مستويات بالغة الخطورة هلك معها الزرع
T	+	-	39	كلما تقدمت السن بالرياضيين فانهم بعامة يجدون ان الاحتفاظ بحالتهم البدنية في مستويات عالية يتزايد صعوبة عاما بعد عام
	+	-	40	بقدر ما اعرف وطبقا للسجلات المحفوظة فان مستوي فضيان النهر لم يبلغ هذا الحد من الارتفاع خلال العشرين سنة الاخيرة
U	+	-	41	حين يكون الاطفال صغارا فانة من السهل العثور علي هدايا ترضيهم ولكن حين يكبرون ويزداد مدى التنوع فيما يحبون ويكرهون يصبح الامر اكثر صعوبة
	+	-	42	ليس من الغريب ان تتباين ميول الناس وان تختلف هوياتهم والالعاب التي يفضلون ممارستها تبعا لمستويات اعمارهم وقدراتهم وتعليمهم وتبعا لتنوع ثقافاتهم وبيئاتهم
(+): Pass		(-): Fail		

Calculation of Raw Score RS	
A- Highest Paragraph Score	
B- Total No. of (-)	
Raw Score RS (A-B)	

صحيفة تحديد المستوى المدخلى

أعلى زوج من الفقرات فى اختبار المفردات

طبق على المفحوص

العمر الزمنى	3.4	5.6	7.8	9.10	11.12	13.14	15.16	17.18	19.20	21.22	23.24	25.26	27.28	29.30	31.32	33.34	35.36	37.38	39.40	41.42	43.44	45.46
2.0 to 2.5	A	A	A	A	B	B	C	C	D	D												
2.6 to 2.11	A	A	A	B	B	B	C	C	D	D	E	E										
3.0 to 3.5	A	A	B	B	C	C	C	D	D	E	E	F	F									
3.6 to 3.11	A	B	B	C	C	D	D	D	E	E	F	F	G	G								
4.0 to 4.5	B	B	C	C	D	D	E	E	E	F	F	G	G	H	H							
4.6 to 4.11	B	B	C	C	D	D	E	E	F	F	G	G	H	H	I							
5.0 to 5.5	B	C	C	D	D	E	E	F	F	F	G	G	H	H	I							
5.6 to 5.11	C	C	D	D	E	E	F	F	G	G	G	H	H	I	I							
6.0 to 6.5	C	C	D	D	E	E	F	F	G	G	H	H	I	I	J							
6.6 to 6.11	C	D	D	E	E	F	F	G	G	H	H	H	I	I	J							
7.0 to 7.5	C	D	D	E	E	F	F	G	G	H	H	H	I	I	J	J						
7.6 to 7.11	C	D	D	E	E	F	F	G	G	H	H	I	I	J	J	K	K					
8.0 to 8.11	C	D	D	E	F	F	G	G	H	H	I	I	J	J	K	K	L	L				
9.0 to 9.11	C	D	D	E	F	G	G	H	H	I	I	J	J	J	K	K	L	L				
10.0 to 10.11	C	D	D	E	F	G	H	H	I	I	J	J	K	K	K	L	L	M	M			
11.0 to 11.11	C	D	D	E	F	G	H	I	I	J	J	K	K	L	L	L	M	M	N	N		
12.0 to 12.11	C	D	D	E	F	G	H	I	I	J	J	K	K	L	L	L	M	M	N	N	O	
13.0 to 13.11	C	D	D	E	F	G	H	I	J	J	K	K	L	L	M	M	M	N	N	O	O	P
14.0 to 14.11	C	D	D	E	F	G	H	I	J	J	K	K	L	L	M	M	N	N	O	O	P	P
15.0 to 15.11	C	D	D	E	F	G	H	I	J	K	K	L	L	M	M	N	N	N	O	O	P	P
16.0 to 16.11	C	D	D	E	F	G	H	I	K	K	L	L	M	M	N	N	O	O	O	P	P	Q
17.0 to 17.11	C	D	D	E	F	G	H	I	K	K	L	L	M	M	N	N	O	O	P	P	Q	Q
18.0 and over	C	D	D	E	F	G	H	I	K	L	L	M	M	N	N	O	O	P	P	P	Q	Q

هذا الجدول هو لتحديد المستوى الدخلى . ويحدد هذا المستوى عند نقطة التقاطع بين العمر الزمنى للمفحوص وأعلى زوج من الفقرات فى إختبار المفردات طبق على المفحوص . فمثلاً ، إذا كان العمر الزمنى للمفحوص اربع سنوات وشهرين (٤ ــ ٢) ، وكان أعلى زوج من الفقرات طبق على المفحوص فى اختبار المفردات هو ١٣ ـ ١٤ ، فإن بقية الاختبارات يبدأ تطبيقها عند المستوى D . ويسجل العمر الزمنى بالسنين والشهور . فإذا كان عمر الطفل هو ٩ سنين وخمسة شهور و١٦ يوماً ، فأن عمره الزمنى المسجل يكون ٩ ــ ٦ . وإذاى كان عمر الطفل ٩ سنين وخمسة شهور و١٥ يوماً ، فأن عمره الزمنى المسجل يكون ٩ ـ ٥ ، تتبع بقية التعليمات كما وردت فى دليل المقياس .

Appendix III

Facial Image Scale (FIS)

Child Name: **Age:** **Gender:** **Address:** **Phone No:**

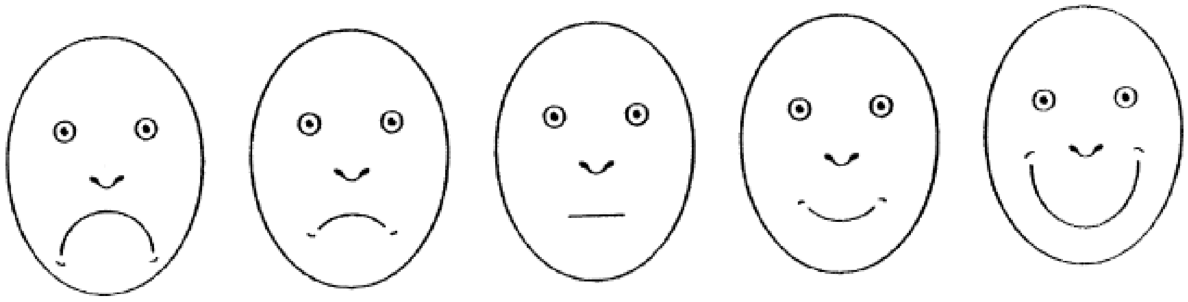

5 **4** **3** **2** **1**

5	4	3	2	1
**Very Unhappy**	**Unhappy**	**Moderate**	**Happy**	**Very Happy**

Appendix IV

Frankl's Behavior Rating Scale (FBRS)

Child No: **Rating:**

Rating			Behavior
1	Rating no. 1	– –	• Refusal of treatment<br>• Forceful crying<br>• Fearfulness,<br>• Or any other overt evidence of extreme negativism.
2	Rating no. 2	–	• Reluctance to accept treatment<br>• Uncooperativeness<br>• Some evidence of negative attitude but not pronounced (sullen, withdrawn).
3	Rating no. 3	+	• Acceptance of treatment<br>• Cautious behavior at times<br>• Willingness to comply with the dentist, at times with reservation, but patient follows the dentist's directions cooperatively.
4	Rating no. 4	++	• Good rapport with the dentist<br>• Interest in the dental procedures<br>• Laughter and enjoyment.
